# Placental lesions in systemic lupus erythematosus: A retrospective histopathological study in a tertiary care center

**DOI:** 10.1097/MD.0000000000051023

**Published:** 2026-10-02

**Authors:** Esra Çobankent Aytekin, Duygu Durna, Havva Serap Toru

**Affiliations:** a Department of Pathology, Konya Numune Hospital, Konya, Turkey; b Department of Pathology, Akdeniz University, School of Medicine, Antalya, Turkey.

**Keywords:** pathology, placenta, pregnancy outcome, premature birth, systemic lupus erythematosus

## Abstract

Systemic lupus erythematosus (SLE) is a chronic inflammatory autoimmune disease of unknown etiology. Pregnancy in patients with SLE, however, poses high-risk factors stemming from both maternal (such as lupus flare and preeclampsia) and fetal (including miscarriage and intrauterine fetal death) complications. Although the pathophysiology underlying obstetric mortality and morbidity in SLE is still under investigation, recent studies have suggested that placental dysfunction may significantly contribute. This study investigated the perinatal outcomes of pregnant women with SLE and the histopathological findings in their placentas. Placenta reports and files from 4820 cases were analyzed between January 2016 and January 2024. A retrospective study of 15 placentas from 11 patients with systemic lupus erythematosus was conducted during this period. We examined 15 placentas from 15 pregnancies in 11 patients with confirmed Systemic lupus erythematosus. The mean maternal age was 28.46 years (range: 20–35). The median gestational age at birth was 36 weeks and 4 days (range: 23 weeks and 4 days to 38 weeks and 3 days), and the median placental weight was 545 g (range: 89.2–828 g). A history of previous pregnancy loss, including abortion or stillbirth, was present in 8 of 11 mothers (72.7%). Two pregnancies that did not receive any treatment during pregnancy resulted in stillbirth. Thirteen cases resulted in live birth. Seven patients were preterm. Five cases had retroplacental hematoma. Our examination revealed fetal vascular malperfusion in 1 case and maternal vascular malperfusion in 8 cases. As a result, the macroscopic and microscopic findings of uteroplacental-vascular pathology and pathologic coagulation in the placentas of patients with SLE are noteworthy.

## 1. Introduction

The placenta serves as an intermediary organ between the mother and the fetus, facilitating fetal nourishment and growth. Placental injury may result in severe pregnancy problems including acute chorioamnionitis, persistent villitis of unknown etiology, maternal vascular malperfusion (MVM), and fetal vascular malperfusion (FVM).^[1,2]^

Systemic lupus erythematosus (SLE) is a chronic inflammatory autoimmune disease of unknown etiology, thought to involve genetic predisposition, environmental triggers, and hormonal effects. SLE is approximately 9 times more common in women than in men and mostly affects women of childbearing age between the ages of 15 and 55 years. Its prevalence in the general female population is approximately 1:1000.^[1]^

SLE is characterized by a heterogeneous pathogenesis and multisystem involvement. It can affect many organ systems, especially the skin and cardiovascular, musculoskeletal, gastrointestinal, hematological, respiratory, urinary, and central nervous systems, and cause serious functional disorders.^[1,3]^ The disease also affects the placental structure during pregnancy, causing complications. SLE, which progresses with remission and exacerbation, can be exacerbated by hormonal changes and physiological adaptations during pregnancy. However, the disease itself can also negatively affect pregnancy through direct antibody-mediated damage to the placenta and maternal organ dysfunction. This 2-way interaction created a vicious cycle.

The incidence of maternal and fetal morbidity and mortality increases in pregnant women with SLE. Pregnancy in these patients is associated with complications, such as miscarriage, premature birth, intrauterine growth retardation (IUGR), placental abruption, and preeclampsia (PE).^[4–6]^ All these complications are primarily related to placental dysfunction. Pharmacological treatment approaches, including hydroxychloroquine (HCQ), low molecular weight heparin (LMWH), aspirin, and steroids, are frequently recommended to prevent these complications.^[5,7]^ Placental developmental disorders are thought to be the basis of adverse pregnancy outcomes in SLE patients. This study investigated the relationship between perinatal outcomes and placental histopathological findings in pregnant women with SLE.

## 2. Materials and methods

In this retrospective study, after obtaining ethics committee approval and data-use permission, we included 15 placentas from 11 pregnant women with confirmed SLE among 4820 placentas evaluated in the pathology department between 2016 and 2024. This study was designed as a retrospective cohort study; however, due to the rarity of pregnancies with SLE, the sample size was small, and the findings should be interpreted accordingly at a descriptive level.

Clinical and obstetric data (such as maternal age, gestational age at delivery, and gestational outcome) were collected from hospital records.

This observational study was conducted in a tertiary care university hospital’s pathology department. The study followed the Declaration of Helsinki and was approved by the Akdeniz University Faculty of Medicine Medical Scientific Research Ethics Committee (Decision no. TBAEK-708, Date: 31.10.2024). The department evaluated 11 consecutive patients diagnosed with SLE among 4820 placentas evaluated between 2016 and 2024.

According to the literature, term, preterm, and postterm definitions were as follows: preterm, <259 days (37 weeks); term: 259 to 293 days (37–41 weeks). Postterm: 294 days (42 weeks) and above.^[8,9]^ Fetuses are classified under Potter-Osanthanondh’s categorization.^[10]^

Small gestational age (SGA): birth weight below the 10th percentile for gestational age. Routine macroscopic examination of the fresh and fixed placentas was performed, considering factors such as weight, size, presence or absence of hematoma, presence of thick-walled and/or tortuous vessels, and presence or absence of grape-like structures. Information on whether it was a single pregnancy or multiple pregnancies was also considered.

Based on the information obtained from the macroscopic examination of the placenta, such as weight, size, presence of hematoma, and infarction, at least 4 parenchymal tissue sections, 2 umbilical cord sections, 1 membrane section, and slides of sections containing the detected pathological lesions were examined. In cases requiring further evaluation, histochemical and immunohistochemical tests were used to establish a definitive diagnosis.

Immunohistochemical staining was performed using an automated Dako immunostainer (Agilent Technologies, Santa Clara). Antigen retrieval was performed using an EDTA buffer (pH 8.0). Primary antibody was applied at a dilution of 1:50 and incubated for 30 minutes at room temperature. Detection was performed using the EnVision Detection System, followed by visualization with diaminobenzidine chromogen and hematoxylin counterstaining.

We used a nephrectomy specimen from a kidney graft diagnosed with acute humoral rejection and normal placental tissue as a positive control for complement degradation product 4d (C4d) staining.^[11,12]^

Hematoxylin and eosin-stained slides, along with immunohistochemical staining, were analyzed using a light microscope (Olympus BX51) by 2 pathologists, one of whom specialized in perinatal pathology, to validate the findings. The pathologists were aware only of the SLE diagnosis and, in 2 cases, the stillbirth outcome, but were otherwise blinded to clinical and treatment data.

Placentas were examined macroscopically and microscopically according to the Amsterdam Placental Workshop Group Consensus Statement and Redline et al.^[13-15]^ The extracted data included macroscopic features such as trimmed placental disc weight and placental weight for gestational age, categorized as small, normal, or large for ≤10th, 11–89th, and ≥90th percentile for gestational age, respectively.^[14]^ These assessments focused on maternal and fetal vascular malperfusion. MVM is defined as the presence of a small placental disk with at least one of the following findings: accelerated villous maturation, placental disk infarction, decidual vasculopathy, or retroplacental/marginal hematoma. FVM is defined by characteristics such as fetal vascular thrombosis, avascular villus, or villous-stromal karyorrhexis. Chorangiosis is characterized by >10 capillaries in >10 terminal chorionic villi in several areas of the placenta.^[16]^

### 2.1. Statistical analysis

Descriptive statistical analysis was performed. Categorical variables were presented as numbers and percentages, while continuous variables were presented as mean ± standard deviation or median (minimum–maximum). Due to the limited sample size and missing data in some subgroups, formal hypothesis testing was not performed, and comparisons were exploratory. Data cleansing, organizing, and analysis were conducted using Microsoft Excel and IBM SPSS Statistics, version 22.0.

## 3. Results

### 3.1. Clinical findings

Out of 4820 placentas retrospectively screened, we analyzed 15 placentas from 15 pregnancies in 11 patients with confirmed SLE. The mean maternal age was 28.46 years (range: 20–35). The median gestational age at birth was 36 weeks and 4 days (range: 23 weeks and 4 days to 38 weeks and 3 days), and the median placental weight was 545 g (range: 89.2–828 g). One patient had lung involvement, but no renal involvement. A history of previous pregnancy loss, including abortion or stillbirth, was present in 8 of 11 mothers (72.7%). HCQ was administered in 11 pregnancies. In 10 pregnancies, the mothers received antiplatelet or anticoagulant therapy. Two pregnancies that did not receive any treatment during pregnancy resulted in stillbirth. Preeclampsia was observed in 3 cases. Thirteen cases resulted in live birth. Seven cases were preterm (<37 weeks), and 8 were term. SGA was observed in 2 infants. No fetal anomalies were observed in any infant. Table 1 summarizes the descriptive findings by treatment status. Table S1, Supplemental Digital Content 1 provides a more detailed breakdown by individual treatment type (hydroxychloroquine, anticoagulant/antiplatelet therapy, corticosteroids, and dual therapy). Antinuclear antibody (ANA) was positive in all cases, and Table 2 shows antibody positivity.

**Table 1:** Clinical and placental pathological findings in the overall cohort and stratified by receipt of any treatment during pregnancy (simplified).

Variable	Overall cohort (n = 15)	Treated (n = 13)	Untreated (n = 2)
Live birth, n (%)	13 (86.7)	13 (100.0)*	0 (0.0)*
Intrauterine growth restriction, n (%)	2 (13.3)	1 (7.7)	1 (50.0)
Preterm birth, n (%)	7 (46.7)	5 (38.5)	2 (100.0)
Preeclampsia, n (%)	3 (20.0)	2 (15.4)	1 (50.0)
Placentomegaly, n (%)	7 (46.7)	7 (53.8)	0 (0.0)
Placental hypoplasia, n (%)	2 (13.3)	1 (7.7)	1 (50.0)
Retroplacental hematoma, n (%)	5 (33.3)	3 (23.1)	2 (100.0)
Pseudocyst, n (%)	1 (6.7)	1 (7.7)	0 (0.0)
Accelerated villous maturation, n (%)	2 (13.3)	1 (7.7)	2 (100.0)
Syncytial knot increase, n (%)	2 (13.3)	1 (7.7)	2 (100.0)
Infarct, n (%)	1 (6.7)	1 (7.7)	0 (0.0)
Intervillitis, n (%)	2 (13.3)	1 (7.7)	1 (50.0)
Deciduitis, n (%)	3 (20.0)	2 (15.4)	1 (50.0)
Chorangiosis, n (%)	6 (40.0)	6 (46.2)	0 (0.0)

“Treated” indicates receipt of at least one of the following during pregnancy: hydroxychloroquine, anticoagulant/antiaggregant therapy, or corticosteroids. “Untreated” indicates no pharmacological treatment during pregnancy.

* Fisher exact test, exploratory analysis given the very small subgroup size (n = 2 untreated); *P* = .01 for live birth. No other statistical comparisons reached significance; given the limited sample size, all percentages should be interpreted descriptively. A detailed breakdown by individual treatment type (hydroxychloroquine, anticoagulant/antiaggregant, corticosteroids, and dual therapy) is provided in Table S1, Supplemental Digital Content 1.

**Table 2:** SLE cohort: clinical, serologic, and placental findings.

Case	Age	Other autoimmune disease	ANA	Anti-Sm/RNP	Anti-SSA	Anti-SSB	Anti-ribosomal P	Lupus anticoagulant antibody	Anti-dsDNA	SGA assessment	Preterm < 37 weeks	Preeclampsia	Live birth	Placental assessment	Syncytiotrophoblast linear C4d	Villous capillary C4d	MVM	FVM
1. case, 1.G	28	None	+	+	−	−	−	−	−	−	+	+	−	N	−	−	+	−
1. case, 2.G	33	RA	+	+	−	−	−	−	−	−	+	−	+	N	−	−	−	−
2. case, 1.G	20	None	+	+	+	+	−	−	+	−	−	−	+	M	+	+	+	−
2. case, 2.G	23	None	+	+	+	+	−	−	+	−	−	−	+	M	−	−	−	−
3. case	24	None	+	−	+	+	−	−	−	−	−	−	+	M	−	−	+	−
4. case, 1.G	31	None	+	+	+	+	+	+	+	−	+	−	−	H	+	−	+	−
4. case, 2.G	32	None	+	+	+	+	+	+	+	−	+	+	+	H	+	−	+	−
5. case	26	None	+	−	+	−	−	−	−	−	−	−	+	M	−	−	−	−
6. case	27	MG	+	−	−	−	−	−	−	+	−	−	+	M	+	−	−	−
7. case	35	None	+	+	−	−	−	−	+	−	+	−	+	M	+	−	+	+
8. case, 1.G	25	None	+	−	+	+	−	−	+	−	−	−	+	N	−	−	−	−
8. case, 2.G	30	None	+	−	+	+	−	−	+	−	−	−	+	N	−	−	+	−
9. case	32	None	+	−	−	−	−	−	+	+	+	+	+	N	+	−	−	−
10. case	32	None	+	−	+	+	−	−	−	−	+	−	+	M	−	−	+	−
11. case	29	None	+	−	+	+	−	−	−	−	−	−	+	N	−	−	−	−

− = negative, + = positive, ANA = antinuclear antibody, C4d = complement degradation product 4d, FVM = fetal vascular malperfusion, G = gravida, H = hypoplasia, M = megaly, MG = myasthenia gravis, MVM = maternal vascular malperfusion, N = normal, RA = rheumatoid arthritis, SGA = small for gestational age.

### 3.2. Gross and histologic findings

Table 3 summarizes gross and histological findings. Placentomegaly was detected in 7 cases, and a hypoplastic placenta in 2 cases. In 6 placentas, the umbilical cord was peripherally located.

**Table 3:** Descriptive histopathological findings of SLE (+) patients in the study.

	n	%		n	%
Placentomegaly	7	46.7	Villous-intervillous-perivillous fibrin accumulation	12	80.0
Placental hypoplasia	2	13.3	Intervillous Thrombosis	0	0.0
Retroplacental hematoma	5	33.3	Accelerated villous maturation	3	20.0
Hydropic villi	3	20.0	Syncytial knots increase	3	20.0
Pseudocyst	1	6.7	Infarct	1	6.7
Mucinous cystic degeneration of the umbilical cord	6	40.0	Chorangiosis	6	40.0
İntervillitis	2	13.3	Deciduit	3	20.0
Villitis	1	6.7	Chorionic vascular ectasia	1	6.7
Avascular villus	0	0.0	FVM finding	1	6.7
MVM finding	8	53.3		0	0.0
Umbilical cord placement					
Central	9	60.0			
Peripheral	6	40.0			
Total	15	100.0		15	100.0

FVM = fetal vascular malperfusion, MVM = maternal vascular malperfusion.

Figures 1 and 2 present representative histopathological findings from 2 illustrative cases, encompassing the spectrum of vascular, inflammatory, and immunohistochemical alterations observed in this cohort. The most common pathological microscopic finding in our study was villous-intervillous-perivillous fibrin accumulation, observed in 80% of cases (Figs. 1C and 2D). Retroplacental hematoma, corresponding microscopically to abruptio placenta, was observed in 33.3% of cases (n = 5; Fig. 2A). Chorangiosis was identified in 40% of cases (Fig. 1A). The single FVM case was histologically characterized by chorionic vascular ectasia (Fig. 1D). Among the 8 cases (53.3%) demonstrating MVM, histopathological correlates included accelerated villous maturation (Fig. 1B, E), hypoplastic villi (Fig. 2B), and increased syncytial knot formation (Fig. 2C). Additionally, histopathological examination demonstrated features of villitis/intervillitis (Fig. 2E) and findings consistent with the amniotic fluid infection sequence (Fig. 2F).

**Figure 1.**
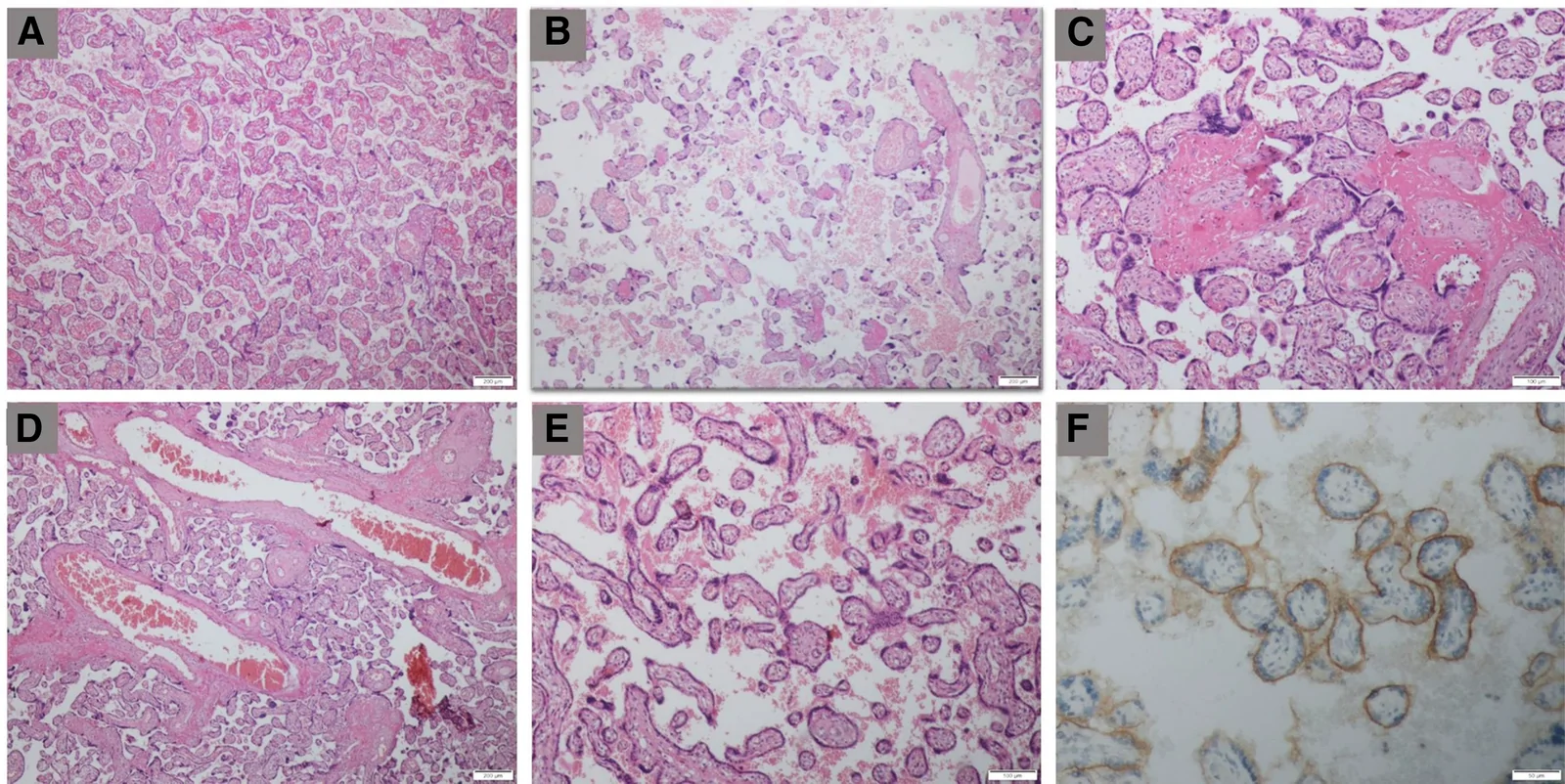
(Patient 6): Placental histopathology from a 35-year-old pregnant woman with a history of stillbirth and recurrent miscarriage. The patient had positive anti-SSA, anti-Sm/RNP, and anti-ribosomal P protein antibodies, as well as normal serum C3 and C4 levels. Lupus anticoagulant was strongly positive. She was receiving hydroxychloroquine and antiplatelet/anticoagulant therapy. The pregnancy resulted in a live birth with IUGR at 27 weeks’ gestation. (A) Chorangiosis H&E X4; (B) accelerated villous maturation H&E X4; (C) villous-intervillous-perivillous fibrin accumulation H&E X20; (D) chorionic vascular ectasia H&E X10; (E) accelerated villous maturation H&E X40; (F) C4d immunohistochemistry X20. C4d = complement degradation product 4d, IUGR = intrauterine growth restriction.

**Figure 2.**
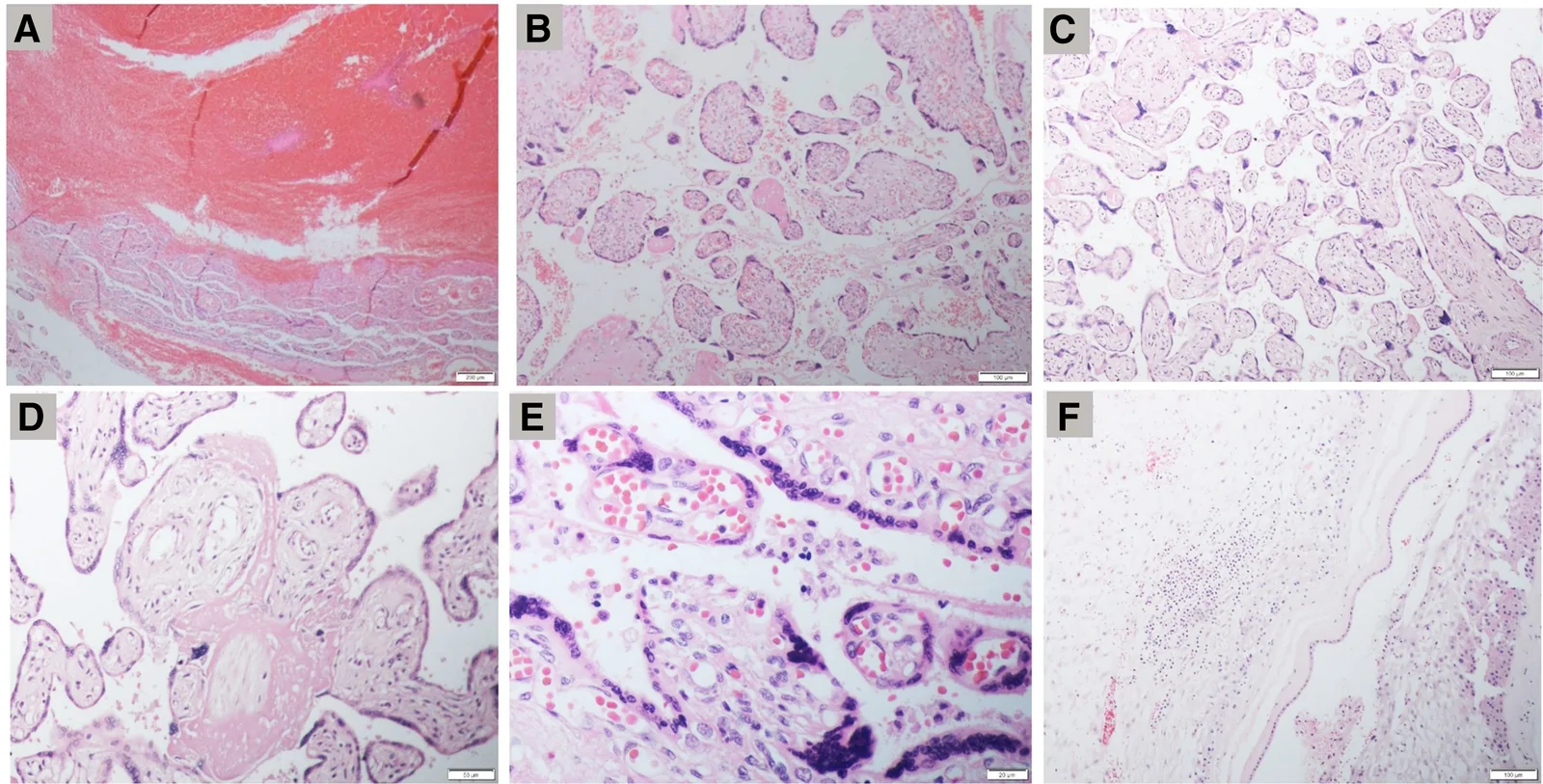
(Patient 1): Histopathological features of the placenta of a 36-year-old pregnant woman with seropositive systemic lupus erythematosus (ANA and anti-Sm/RNP positive), elevated serum C3 levels, and no clinical organ involvement. The patient had not received any treatment. In the 25th week of pregnancy, abruptio placenta developed and resulted in stillbirth. (A) Abruptio placenta microscopic appearance. H&E X40; (B) hypoplastic villi H&E X20; (C) increased syncytial knots appearance H&E X10; (D) villous-intervillous-perivillous fibrin accumulation H&E X20; (E) villitis/intervillitis H&E X40; (F) amniotic fluid infection sequence H&E X10. ANA = antinuclear antibody.

### 3.3. Immunohistochemical findings

C4d was identified in 6 of the 15 placentas (40%) from women with SLE. All C4d-positive cases exhibited maternal vascular malperfusion (MVM). When present, C4d appeared as a focal or diffuse membranous and cytoplasmic staining pattern in syncytiotrophoblasts (Fig. 1F). Positive staining for vascular endothelial cells was detected in only 1 case.

## 4. Discussion

The placenta remains a largely enigmatic organ that develops during pregnancy. Placental pathology serves as a primary approach for investigating the causes of adverse pregnancy outcomes. SLE is a multisystem disease that can lead to obstetric complications by directly affecting the placenta and by exacerbating maternal disease activity, resulting in dysfunction of other organ systems.

Two important methodological limitations must be considered in interpreting this study. First, although a comparison group consisting of pregnancies without SLE was also included in the design of this study, statistical comparisons between the groups were not presented in this analysis due to the limited sample size of both groups; therefore, it was not possible to directly evaluate whether the observed placental lesions were specific to SLE within the scope of this study. Second, placentas from all pregnancies delivered at our institution are routinely sent for pathological evaluation. The fact that the study was conducted in a tertiary university hospital increases the likelihood that pregnant women with a known SLE diagnosis will more frequently seek or be referred to such centers. However, some patients in our cohort had not yet received an SLE diagnosis during pregnancy, and the SLE diagnosis was made during or after evaluations for pregnancy complications. Therefore, the cohort consists not only of patients referred due to a known SLE diagnosis, but also of patients who presented due to pregnancy complications and were subsequently diagnosed with SLE; this heterogeneous structure makes it difficult to assess the direction and magnitude of possible selection bias. Therefore, caution should be exercised in directly generalizing reported prevalence rates (e.g., MVM, PVFD, C4d positivity) to the entire pregnant population with SLE.

A history of previous pregnancy loss, including abortion or stillbirth, was present in 8 of 11 mothers (72.7%). Phuti et al (2020) similarly revealed that unfavorable pregnancy outcomes, including miscarriages, early births, and infertility, frequently occur prior to the diagnosis of SLE, with only a minority of individuals attaining uncomplicated full-term deliveries.^[17]^ These data indicate that SLE may adversely impact reproductive health even during the clinically undiagnosed phase.

In our study, we observed negative pregnancy outcomes. Preterm delivery (<37 weeks) was observed in 7 cases (46.7%), and IUGR was recognized in 2 infants (13.3%). Moreover, fetal death was noted in 2 instances (13.3%). Similarly, the literature indicates that common fetal problems include spontaneous abortion (16.0%), preterm delivery (39.4%), neonatal mortality (2.5%), and IUGR (12% exhibiting IUGR).^[18]^ These findings underscore the persistent risk of considerable maternal and perinatal problems in pregnancies affected by SLE, consistent with prior literature descriptions. Preterm birth in SLE may result from clinical or subclinical inflammation, autoantibodies, hormone dysregulation, lupus-related immunological anomalies, or pharmacological treatments aimed at managing disease activity. The risk increases when disease activity is evident at the beginning of pregnancy and organ involvement occurs.^[19,20]^ In a 2015 study by Jakobsen et al, 62 of 84 SLE pregnancies resulted in live births; PE was detected in 8.3% of the cases, and the rates of premature birth (*P* = .0032), cesarean section (*P* = .015), and IUGR (*P* = .003) were reported to be significantly higher. The study also reported the loss of 2 babies following an extremely premature birth.^[21]^ In our study, PE was detected in 3 cases, representing 20% of the population; this rate was significantly higher than the 8.3% rate reported by Jakobsen et al in 2015. This difference may be attributed to established PE risk factors in the sample, such as ANA and anti-Sm/RNP positivity in 1 case; concurrent lupus anticoagulant, ANA, and anti-Sm/RNP positivity in another case; and lupus anticoagulant positivity in a third patient.^[18,19,21]^ Studies in the literature indicate that pregnancy loss rates are higher in pregnant women with SLE than in healthy pregnant women, particularly in cases with organ involvement. Furthermore, the presence of renal or neurological involvement, along with anti-Ro/SSA, anti-La/SSB, anti-thyroglobulin, and especially antiphospholipid antibody (aPL) positivity, has been reported as significant factors that increase the risk of fetal loss.^[22]^ In our study, fetal loss occurred in 2 of 15 cases (13.3%). The first patient tested positive for ANA, anti-Sm/RNP, Ku, and Mi-2 antibodies, while the second patient was positive for anti-Ro/SSA, anti-Sm/RNP, and antiphospholipid antibodies. Both patients had not yet been diagnosed with SLE and consequently did not receive any treatment during pregnancy. These 2 women also had their first pregnancies included in our study. Although the mothers had the same antibody positivity and clinical condition as in their previous pregnancies, they received different treatments with HCQ, anticoagulants/antiplatelets, and steroids in subsequent pregnancies. Both pregnancies, 1 preterm and the other at term, resulted in live births. Despite the limited sample size, the observation that both mothers experienced fetal loss in their first untreated pregnancies, followed by live births in subsequent treated pregnancies, represents a significant finding of this study. This observation aligns with existing literature, which indicates that appropriate immunosuppressive and antithrombotic treatment strategies can enhance obstetric outcomes in pregnant women with SLE.^[1,23,24]^ However, given the very limited number of cases, this observation should be interpreted not as independent evidence of treatment efficacy, but as supporting the existing evidence of treatment benefit in SLE pregnancies. These pregnancy complications affecting both the mother and fetus share a common histopathology, including abnormal uteroplacental vascular transformation, impaired placentation, and evidence of chronic inflammation.^[1,7,21]^

In our study, atrioventricular (AV) block was detected in 2 patients across 3 pregnancies. Anti-Ro SSA, anti-Sm/RNP, and anti-ribosomal P protein antibodies were positive in both patients. A retrospective study by Krishnan et al reported that, in addition to congenital heart block, structural cardiac anomalies may also be observed in fetuses of mothers with serological or clinical evidence of systemic lupus erythematosus (SLE).^[25]^ In particular, anti-Ro/SSA and anti-La/SSB antibodies have been strongly associated with congenital heart block, crossing the placental barrier after 16 weeks of gestation, regardless of maternal disease activity or classification.^[25,26]^

Thus, both our findings and the literature indicate that the presence of autoantibodies in mothers with SLE is a critical risk factor for fetal cardiac morbidity.^[25,26]^ AV block was identified in 3 instances in our investigation, comprising 2 cases with positive anti-Ro/SSA and anti-La/SSB antibodies and 1 case with positive anti-dsDNA antibodies.

Hydroxychloroquine is widely used for the treatment of SLE owing to its anti-inflammatory and immunomodulatory properties. HCQ has been shown to reduce the incidence of obstetric complications such as maternal lupus flares, neonatal lupus syndrome, preterm birth, fetal growth restriction, and PE.^[23]^ Furthermore, aspirin, by regulating the balance of prostacyclin and thromboxane, contributes to improved early placentation and increased uteroplacental blood flow. It can also reduce the risk of preterm pre-eclampsia, which is thought to be a major cause of poor placentation, by 90%. Current Dutch obstetric guidelines recommend the use of LMWH in combination with aspirin in women with SLE. Indeed, studies have reported that heparin and aspirin are effective in preventing coagulation-related lesions, which play a role in SLE pathogenesis.^[23,27]^

Studies on placental lesions in SLE patients are limited. Redline and Abramowsky^[28]^ and Labarrere et al.^[29]^ were pioneers in systematically documenting elevated rates of chronic villitis and uteroplacental vascular lesions in SLE, emphasizing that the condition may adversely affect placental circulation at both maternal and fetal levels. Subsequent investigations have shown analogous findings, predominantly involving vascular anomalies and coagulation problems (e.g., infarction and decidual thrombi), alongside chronic inflammation.

Some studies suggest that villous lesions with uteroplacental vascular pathology develop secondary to physiological changes in the myometrial segment of spiral arteries, which may reflect aberrant recognition of trophoblastic antigens by the immune system.^[26]^

Previous studies have documented increased rates of maternal and fetal vascular malperfusion in pregnancies complicated by systemic lupus erythematosus (SLE). Maternal vascular malperfusion (MVM) indicates reduced maternal blood flow and results in characteristic alterations in placental growth and distal villous morphology.^[15]^ In our study, MVM findings were detected in 53.3% (8/15) of cases, while FVM findings were observed in only 1 case (6.7%). This significant difference is thought to be due to the fact that placental pathology in our sample predominantly indicated maternal perfusion impairment, and fetal-side vascular thrombotic lesions (such as intravillous thrombosis and avascular villi) were rare in the cases included in the study. This is consistent with the reporting of MVM being more prevalent than FVM in SLE series in the literature. The high prevalence of MVM in our cohort may reflect systemic endothelial dysfunction. In vitro studies on endothelial cells exposed to sera from pregnancies complicated by intrauterine growth restriction and PE have shown decreased endothelial viability, increased oxidative stress, and mitochondrial damage (mitochondrial swelling, autophagic vacuole formation) after prolonged exposure; these changes have been associated with decreased vascular endothelial growth factor (VEGF) levels.^[30]^ While these findings suggest that endothelial and mitochondrial mechanisms may underlie MVM, this relationship remains an indirect inference because this study did not include direct ultrastructural examination of the endothelium.

Dhita et al reported maternal vascular malperfusion (MVM) and fetal vascular malperfusion (FVM) in 83% and 33% of small for gestational age (SGA) infants with systemic lupus erythematosus (SLE), respectively. Among SGA patients without SLE, the rates were 64% for MVM and 39% for FVM. The study also identified a higher prevalence of perivillous fibrin deposition (PVFD) in the SLE group.^[6]^ Similarly, Salafia and Parke emphasized the importance of evaluating placental thrombotic lesions and perivillous fibrin deposition. They stated that although some fibrin deposition occurs even in normal pregnancies, increased PVFD should be considered pathological.^[31]^ PVFD was the most common histopathological finding in our study, supporting this finding.

Previous studies indicate that placental weight is typically reduced and hypoplasia occurs more frequently in pregnant women with systemic lupus erythematosus (SLE). Contributing factors to lower placental weight in SLE cases, compared to healthy pregnancies, include increased disease activity, thrombocytopenia, and hypocomplementemia.^[32]^ Low placental weight is a significant indicator of maternal malperfusion and a predictive factor for stillbirth.^[13]^ Placental hypoplasia was identified in 2 placentas (patient 4; associated with MVM). This finding may reflect the absence of clinical conditions typically associated with hypoplasia or the use of preventive treatments. Alternatively, increased placental weight due to edematous changes in the chorionic mesenchyme, increased perivillous fibrin deposition, or chorangiomatosis may explain this observation.^[33]^ This comment remains speculative, and further studies are needed to clarify the mechanisms causing placentomegaly in SLE pregnancies.

Choriangiosis is a histopathological change caused by prolonged low-grade hypoxia in placental tissue. Chorangiosis was the second most common microscopic pathological finding in our study, and this lesion likely reflected a compensatory mechanism secondary to inadequate oxygen transfer and MVM.

Retroplacental hematoma was identified in 46.7% of cases (n = 7), exceeding previously reported rates. The incidence was higher among patients treated with LMWH. However, as no formal statistical analyses or adjustments for confounding variables were conducted, this observation remains exploratory and should be regarded as hypothesis-generating rather than evidence of a causal relationship.^[6]^

We detected signs of acute and chronic inflammation in the villous/intervillous areas (n = 2) and the decidua (n = 3) in 5 placentas. Chronic inflammatory lesions may also reflect vasculitis, a common feature of SLE.^[26]^ In the literature, pathological findings such as villitis and intervillitis have been reported in SLE pregnancies; however, these lesions are not specific to SLE and have been described in a variety of clinical settings. They have been associated with adverse fetal and maternal outcomes.^[1,6]^ In our study, anti-inflammatory treatments, particularly HCQ, may have contributed to the lower detection rate of these lesions.

In our study, 40% (n = 6) of cases showed positive C4d staining in syncytiotrophoblasts. Additionally, positive staining was seen in vascular endothelial cells in only 1 case, which was evaluated in favor of neonatal SLE and noted in the report.^[12]^ All cases with C4d accumulation also showed PVFD and MVM findings. Although the mechanisms leading to PVFD are not fully understood, immune dysregulation, including complement activation, is thought to play a significant role. The role of complement activation in this process is supported by the C4d positivity observed in placentas with increased PVFD.^[34,35]^

The complement system plays a physiological role in normal placentation: C1q, produced by extravillous trophoblasts, contributes to trophoblast invasion and decidual spiral artery remodeling, while over- or dysregulated activation of the classical pathway – reflected by tissue-level C4d accumulation and, at a more advanced stage, C5b-9 – has been associated with villous damage and adverse pregnancy outcomes in SLE and antiphospholipid syndrome.^[1]^ While the co-occurrence of C4d positivity with PVFD and MVM in our findings is consistent with this mechanism, the small sample size and lack of a control group prevent any definitive conclusions about the diagnostic or predictive value of C4d in this context. Larger, controlled studies including additional complement activation markers (e.g., C3b, C5b-9) are needed before C4d can be considered a candidate clinically viable biomarker.

Similarly, studies have reported increased placental C4d accumulation in patients with SLE and positive aPL, which is associated with low placental weight, low birth weight, and earlier delivery. Our findings are broadly consistent with this, as C4d positive staining was observed in all SGA cases, 57.1% of preterm deliveries, 62.5% of PVFD cases, and 50% of placentas with MVM-related findings. These findings may suggest an association between C4d positivity and adverse pregnancy outcomes; however, given the limitations noted above – particularly the small sample size and lack of a control group – this observation should be treated as preliminary and hypothesis-generating rather than evidence supporting a defined biomarker role.^[6]^ Consequently, complement-targeted or immunosuppressive treatment strategies may warrant further investigation in managing SLE-related pregnancy complications; however, as mentioned above, larger, more controlled studies are needed before C4d can be evaluated as a standalone biomarker.^[11]^ Consequently, complement-targeted or immunosuppressive treatment strategies may warrant further investigation for managing SLE-related pregnancy complications; however, as noted above, larger, more controlled studies are needed before C4d can be evaluated as a standalone biomarker.

Beyond their known association with thrombosis, antiphospholipid antibodies (aPLs) may also directly contribute to trophoblastic damage. Anti-β2GPI antibodies have been reported to bind to trophoblasts and decidual endothelial cells, disrupting VEGF production, suppressing extravillous trophoblast invasion, and reducing annexin V expression, thereby predisposing to placental infarction and recurrent pregnancy loss.^[1]^ In our cohort, lupus anticoagulant positivity was detected in only 1 patient (patient 4) in both pregnancies, and both placentas of this patient showed C4d positivity, MVM, and placental hypoplasia. The first pregnancy, which did not receive treatment, resulted in fetal loss, while the second pregnancy, which received treatment, resulted in live birth. Furthermore, C4d positivity was significantly more frequent in anti-dsDNA positive cases (62.5%; 5/8) compared to anti-dsDNA negative cases (14.3%; 1/7). This finding is consistent with the hypothesis that complement-binding autoantibodies may trigger classical pathway activation at the placental level. However, the very limited number of aPL-positive cases in our cohort (two pregnancies in only 1 patient) limits the generalizability of this relationship and requires confirmation in larger studies.

Patients with SLE are at high risk for adverse pregnancy outcomes due to inflammation, autoantibody production, and hormonal imbalances that affect placental function. A better understanding of placental pathology is crucial to preventing, predicting, and treating adverse perinatal outcomes. The vascular pathologies and coagulation disorders observed in the placenta support the pathophysiological basis of this effect. A more detailed examination of the effects of SLE on the placenta will contribute to developing treatment strategies, reducing complications, and improving pregnancy outcomes. Furthermore, careful pregnancy planning, close management, and appropriate treatment can significantly reduce these risks. Our study revealed various pathological findings associated with immunological mechanisms in the placentas of SLE patients. The proposed relationships between SLE, complement activation, endothelial damage, placental vascular pathology, and adverse pregnancy outcomes are schematically summarized in Figure 3.

**Figure 3.**
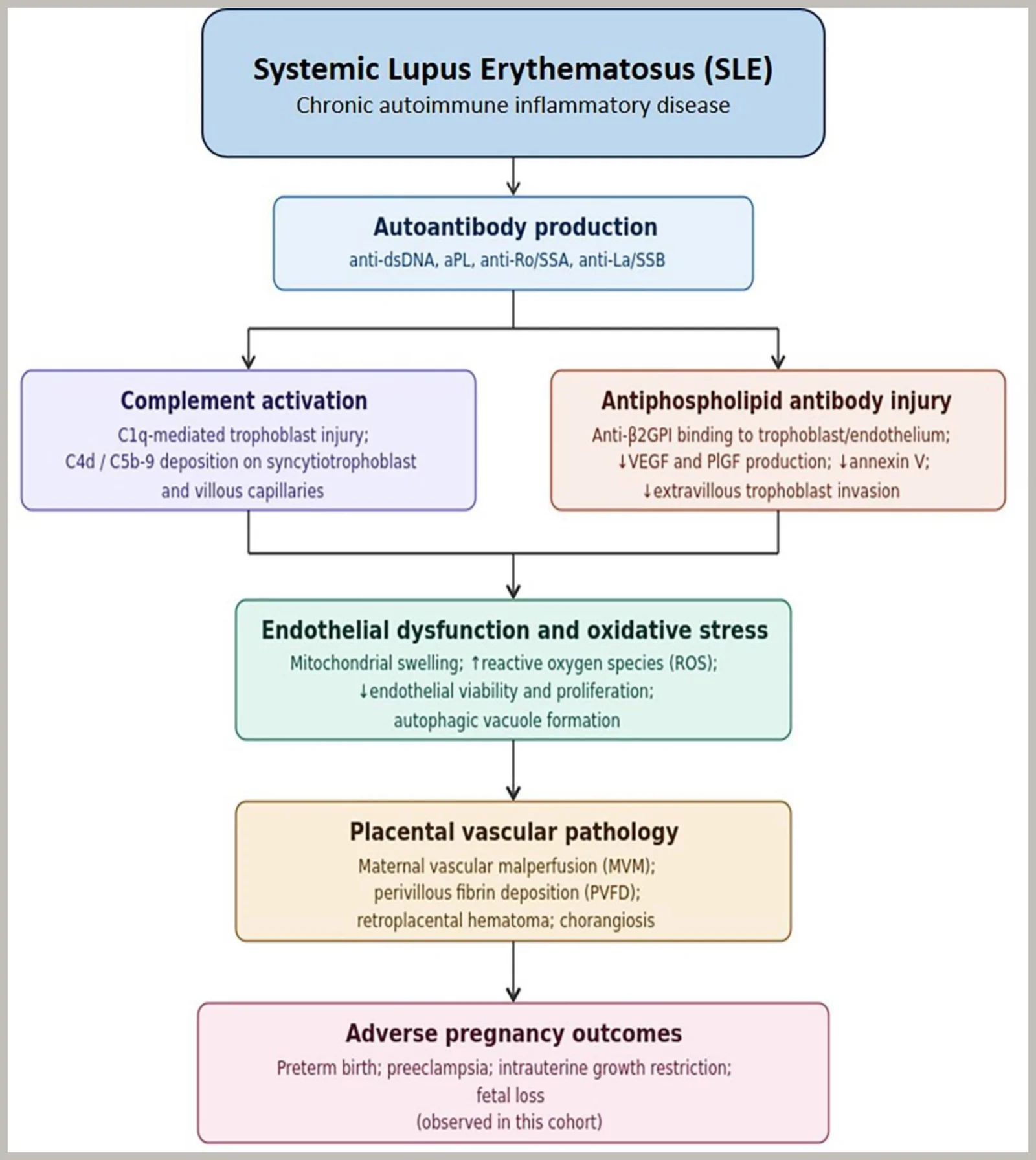
The proposed mechanistic pathway linking SLE to adverse pregnancy outcomes. This diagram summarizes the relationships suggested in the literature (complement activation and antiphospholipid antibody-mediated mechanisms, endothelial and mitochondrial dysfunction); these relationships may link SLE to placental vascular pathology and pregnancy complications observed in this cohort. The pathway is derived from previous mechanistic studies (References 1 and 35); direct causal links were not tested in this study, and the diagram should be interpreted as a conceptual framework rather than a proven sequence of events. aPL = antiphospholipid antibody, C4d = complement degradation product 4d, MVM = maternal vascular malperfusion, PVFD = villous-intervillous-perivillous fibrin deposition, ROS = reactive oxygen species, SLE = systemic lupus erythematosus, VEGF = vascular endothelial growth factor.

As a result, the macroscopic and microscopic findings of uteroplacental-vascular pathology and pathologic coagulation in the placentas of patients with Systemic Lupus Erythematosus are noteworthy. Because few studies have examined placental pathology in SLE pregnancies, our study makes a significant contribution to the literature.

The main limitations of the study are as follows:

- The limited sample size (15 placentas from 11 patients) reduces the statistical power of the analyses, especially for those based on treatment subgroups.
- Although a comparison group of pregnancies without SLE was identified, it was not included in the analysis due to the small sample size. As a result, the specificity of the observed lesions to SLE could not be directly evaluated.
- Because all placentas from births at the institution are routinely sent for pathological evaluation, selection bias in placenta sampling is minimized. However, as the study was conducted in a tertiary care center, patients with a known diagnosis of SLE may be more likely to seek treatment at such facilities. Additionally, some patients in the cohort were diagnosed with SLE after pregnancy complications rather than prior to pregnancy. These factors complicate the assessment of potential selection bias; thus, the findings may not be generalizable to all pregnancies with SLE.

Due to the small sample size, statistical adjustment for potential confounding variables (such as maternal age, disease activity, and treatment combinations) was not feasible. Therefore, the findings should be regarded as descriptive and hypothesis-generating.

## Acknowledgments

The authors have reviewed and edited the manuscript and take full responsibility for the content of this publication.”

## Author contributions

**Conceptualization:** Esra Çobankent Aytekin, Havva Serap Toru.

**Data curation:** Esra Çobankent Aytekin, Duygu Durna, Havva Serap Toru.

**Formal analysis:** Esra Çobankent Aytekin, Duygu Durna, Havva Serap Toru.

**Investigation:** Esra Çobankent Aytekin, Havva Serap Toru.

**Methodology:** Esra Çobankent Aytekin, Havva Serap Toru.

**Project administration:** Havva Serap Toru.

**Resources:** Esra Çobankent Aytekin, Havva Serap Toru.

**Supervision:** Havva Serap Toru.

**Validation:** Havva Serap Toru.

**Visualization:** Esra Çobankent Aytekin, Havva Serap Toru.

**Writing – original draft:** Esra Çobankent Aytekin.

**Writing – review & editing:** Esra Çobankent Aytekin, Havva Serap Toru.


