# Supplementary material for: Placental lesions in systemic lupus erythematosus: A retrospective histopathological study in a tertiary care center
Source: Medicine (Baltimore). 2026 Oct 2;105(40):e51023. doi: 10.1097/MD.0000000000051023 (PMC13633014; doi:10.1097/MD.0000000000051023)
Supplement: Supplementary file 1 [file medi-105-e51023-s001.docx]

**Supplementary Table S1: Descriptive findings according to treatment status**

|  |  | Live Birth | Intrauterine growth restriction | Preterm | Preeclampsia | Placentomegaly | Placental Hypoplasia | Retroplacental Hematoma | Pseudocyst | Accelerated Villous Maturation | Syncytial knots Increase | Infarct | İntervillitis | Deciduit | Chorangiosis |
| --- | --- | --- | --- | --- | --- | --- | --- | --- | --- | --- | --- | --- | --- | --- | --- |
|  |  | n(%) | n(%) | n(%) | n(%) | n(%) | n(%) | n(%) | n(%) | n(%) | n(%) | n(%) | n(%) | n(%) | n(%) |
| **Hydroxychloroquine (HCQ) treatment** | Y | 11(100.0) | 1(9,1) | 5(45,5) | 2(18,2) | 6(54,5) | 0(0,0) | 3(27,3) | 0(0,0) | 1(9,1) | 1(9,1) | 1(9,1) | 1(9,1) | 2(18,2) | 6(54,5) |
|  | N | 2(50,0) | 1(25,0) | 2(50,0) | 1(25,0) | 1(25,0) | 1(25,0) | 2(50,0) | 1(25,0) | 0(0,0) | 1(25,0) | 0(0,0) | 1(25,0) | 1(25,0) | 0(0,0) |
| **Anticoagulant / Antiaggregant** | Y | 9(100.0) | 1(11,1) | 4(44,4) | 1(11,1) | 4(44,4) | 0(0,0) | 1(11,1) | 0(0,0) | 1(11,1) | 1(11,1) | 1(11,1) | 0(0,0) | 1(11,1) | 4(44,4) |
|  | N | 4(66,7) | 1(16,7) | 3(50,0) | 2(33,3) | 3(50.0) | 1(16,7) | 4(66,7) | 1(16,7) | 0(0,0) | 1(16,7) | 0(0,0) | 2(33,3) | 2(33,3) | 2(33,3) |
| **Steroid Therapy** | Y | 4(100,0) | 0(0,0) | 2(50,0) | 1(25,0) | 2(50,0) | 0(0,0) | 0(0,0) | 1(25,0) | 0(0,0) | 0(0,0) | 0(0,0) | 0(0,0) | 0(0,0) | 1(25,0) |
|  | N | 9(81,8) | 2(18,2) | 5(45,5) | 2(18,2) | 5(45,5) | 1(9,1) | 5(45,5) | 0(0,0) | 1(9,1) | 2(18,2) | 1(9,1) | 2(18,2) | 3(27,3) | 5(45,5) |
| **Dual Therapy** | Y | 9(100,0) | 1(11,1) | 5(55,6) | 2(22,2) | 4(44,4) | 0(0,0) | 1(11,1) | 0(0,0) | 1(11,1) | 1(11,1) | 1(11,1) | 0(0,0) | 1(11,1) | 5(55,6) |
|  | N | 4(66,7) | 1(16,7) | 2(33,3) | 1(16,7) | 3(50,0) | 1(16,7) | 4(66,7) | 1(16,7) | 0(0,0) | 1(16,7) | 0(0,0) | 2(33,3) | 2(33,3) | 1(16,7) |
| **Any Treatment** | Y | 13(100,0)^*^ | 1(7,7) | 5(38,5) | 2(15,4) | 7(53,8) | 0(0,0) | 3(23,1) | 1(7,7) | 1(7,7) | 1(7,7) | 1(7,7) | 1(7,7) | 2(15,4) | 6(46,2) |
|  | N | 0(0,0) | 1(50,0) | 2(100,0) | 1(50,0) | 0(0,0) | 1(50,0) | 2(100.0) | 0(0,0) | 2(100.0) | 2(100.0) | 0(0,0) | 1(50,0) | 1(50,0) | 0(0,0) |

Fisher Exact Test, *P=0,01 Y:Yes N: No

Data are presented as n(%). IUGR: intrauterine growth restriction; HCQ: hydroxychloroquine; Y: Yes (treatment received); N: No (treatment not received). Owing to the very small subgroup sizes in this granular breakdown, no formal statistical testing was performed for these comparisons; findings should be interpreted descriptively and as hypothesis-generating. The simplified overall/treated/untreated comparison (including the single statistically notable finding, Fisher's exact test for live birth vs. any treatment, p = 0.01) is presented in the main-text Table 1.
